# Prediction of microseismic events in rock burst mines based on MEA-BP neural network

**DOI:** 10.1038/s41598-023-35500-1

**Published:** 2023-06-12

**Authors:** Tianwei Lan, Xutao Guo, Zhijia Zhang, Mingwei Liu

**Affiliations:** Liaoning Technical University, Fuxin, China

**Keywords:** Natural hazards, Solid Earth sciences

## Abstract

Microseismic monitoring is an important tool for predicting and preventing rock burst incidents in mines, as it provides precursor information on rock burst. To improve the prediction accuracy of microseismic events in rock burst mines, the working face of the Hegang Junde coal mine is selected as the research object, and the research data will consist of the microseismic monitoring data from this working face over the past 4 years, adopts expert system and temporal energy data mining method to fuse and analyze the mine pressure manifestation regularity and microseismic data, and the "noise reduction" data model is established. By comparing the MEA-BP and traditional BP neural network models, the results of the study show that the prediction accuracy of the MEA-BP neural network model was higher than that of the BP neural network. The absolute and relative errors of the MEA-BP neural network were reduced by 247.24 J and 46.6%, respectively. Combined with the online monitoring data of the KJ550 rock burst, the MEA-BP neural network proved to be more effective in microseismic energy prediction and improved the accuracy of microseismic event prediction in rock burst mines.

## Introduction

One of the worst accidents that can happen during the mining of coal is a rock burst. Therefore, the ability to foresee the occurrence of rock burst events is crucial. Microseismic monitoring is an important technical tool to obtain information on the precursors of rock burst. The advanced anticipation of microseismic monitoring information is particularly important.

Taking into account the seismicity, magnitude (energy), and frequency of a region, it can directly reflect the "enhancement" or "calmness" of seismic activity quantitatively^1^. This shows that the rock burst is often accompanied by the occurrence of microseismic events before the occurrence of the rock burst, and the sudden change of energy and frequency can be regarded as a sign of rock burst danger^2^.

Zhang et al.^3^ investigated the regional hazard prediction of rock burst in deep mining by microseismic energy attenuation laminar imaging. The study shows that regional rock burst prediction using microseismic energy attenuation is an effective way to reveal the characteristics of rock burst. Hang Zhang and Jun Zeng et al. used neural networks for multi-microseismic parameter time series prediction starting from the microseismic time series of rock burst. It provides a good idea for microseismic magnitude (energy) time series periodicity prediction^4^.

Research on the theory and application of artificial neural networks has been re-emerging worldwide since the mid-1980s^5^. Xie et al.^6^ proposed a study based on Bagging-SVM to investigate the role of multi-source microseismic data in rock burst hazard prediction, and Li et al.^7^ proposed a Bayesian network-based dynamic early warning of microseismic multiparameter rock burst. Both of them demonstrate the feasibility of neural networks in microseismic prediction applications. Hui Liu, Jiulong Cheng, et al. investigated microseismic intensity prediction by radial-based probabilistic neural networks, exploring the sample process characteristics and control of the regularity of microseismic and periodically weighted events, and studying the statistical regularity, trends, and critical features behind the event sample data to predict the microseismic magnitude and risk level^8^.

Chen and Pan^9^ also proposed the application of the BP algorithm with genetically simulated annealing in rock burst for the prediction study of earthquake magnitude (energy). The prediction results are influenced by factors such as the variability of the algorithm structure and the different ways of data processing, while the thinking evolutionary algorithm has unique advantages over genetic algorithms, such as inherent structural parallelism, high overall search efficiency, etc., which helps to improve the accuracy of prediction. As a result, the BP neural network optimized by the evolutionary thinking algorithm may be superior to the BP neural network optimized by the genetic algorithm.

Wang et al.^10^ investigated the evolutionary and generalization ability and predictability of the MEA-BP neural network and applied it to sea wave height warning and applied it to the wave height warning. Yu et al.^11^ applied the MEA-BP neural network model to the standard friction prediction of long-distance hot oil pipelines, and their predictions showed that the MEA-BP model prediction results have high accuracy and small dispersion. Chen and Laghrouche^12^ applied the MEA algorithm and PSO algorithm in the proton exchange membrane fuel cell. The evolutionary algorithms including the MEA algorithm, PSO algorithm, and genetic algorithm (GA) were applied to optimize the parameters of the established proton exchange membrane fuel cell aging prediction model in the aging prediction of the proton exchange membrane fuel cell (PEMFC). The results showed that the parameter accuracy optimized by the MEA algorithm was improved by 10 times. Zhang et al.^13^ applied the MEA-BP neural network to predict the mechanical parameters of the surrounding rocks in the Pingdingshan mine and proved that the MEA-BP algorithm could improve the accuracy of the prediction. Yang et al.^14^ used the vegetation of the abandoned land in the arid mine. The results of training the MEA-BP algorithm using the vegetation cover and biomass maps of the abandoned land in the arid mine area also showed the advantages of the algorithm.

The MEA-BP neural network method is used to predict microseismic energy in light of the study above, and the new characteristics of the MEA algorithm are used to optimize the BP neural network. The MEA algorithm’s primary operating premise is that it adjusts the weights and thresholds of the BP neural network to prevent locally optimum solutions and too poor convergence during training.

## MEA-BP neural network prediction model construction

### "Period-energy-frequency" data model construction and data processing

**Figure 1:**
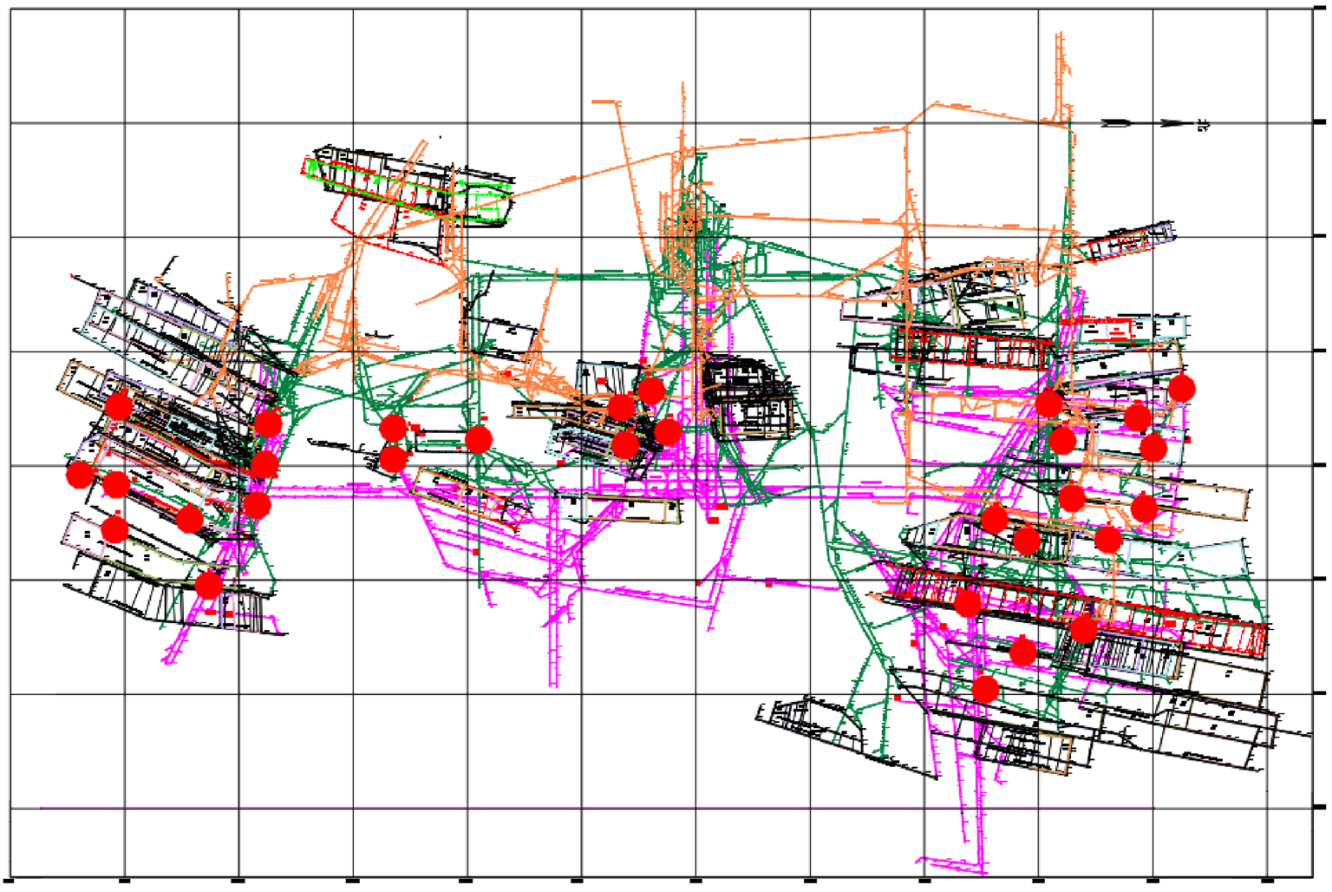
SOS micro-earthquake monitoring system.

Junde coal mine is equipped with an SOS micro-earthquake monitoring system, and its simple layout is shown in Fig. 1.

The data of a horizontal working face from November 2017 to August 2021 were obtained, and 30 sets of data were collected each month, and a total of 910 sets of valid data were collected, and each set of data was divided into accumulated energy, maximum energy, average energy, and frequency. At present, many coal mines in China regard the microseismic large energy events concentrated in a short time as the precursor of rock burst.

Zhao et al.^15^ analyzed the regularity of energy accumulation and release of coal seam roof under the influence of working face retrieval speed, mainly studied the regularity of roof energy accumulation under the effect of retrieval speed, then analyzed the characteristics of roof energy release, and finally explored the mechanism of roof energy accumulation and release under the influence of retrieval speed. Zhang et al.^16^ studied the microseismic regularity during the deep shaft roadway crossing fault group, and Zhen et al.^17^ analyzed the impact damage energy characteristics of the surrounding rock in the back mining roadway, which mainly studied the energy characteristics of the coal rock body when the impact damage occurred based on the microseismic monitoring energy characteristics of the typical tunnel impact damage, and carried out the conventional triaxial compression test and combined with theoretical analysis and numerical simulation. Li et al.^18^ studied the change process of microseismic parameters such as microseismic event rate, energy release, apparent volume, energy index, Schmidt number, b-value, and seismic response coefficient and their relationship with surrounding rock damage. Zhang et al.^19^ analyzed the change in spatial correlation length of microseismic events in mines at different spatial scales and explained the mechanism behind it. Wang et al.^20^ studied the spatial and temporal distribution and evolution of microseismic events and analyzed the differences and correlations of multi-coal mining microseismic events under high-location thick and hard rock layers, especially the relationship between high-energy mining earthquakes and fractures and the motion pattern of high-location thick and hard rock layers. Ding^21^ analyzed the evolution of mining seismic time sequence under different coal mining methods in the Huayan coalfield^.^ There are two main methods of microseismic early warning methods for rock burst. One is the method of early warning when the microseismic frequency, total microseismic energy, or the maximum value of microseismic energy reaches or exceeds the critical warning value. The other is the method of early warning when there is a continuous increase in microseismic frequency and total microseismic energy, an abnormal change in microseismic frequency and total microseismic energy, and the accumulation of microseismic events in a local area.

**Figure 2:**
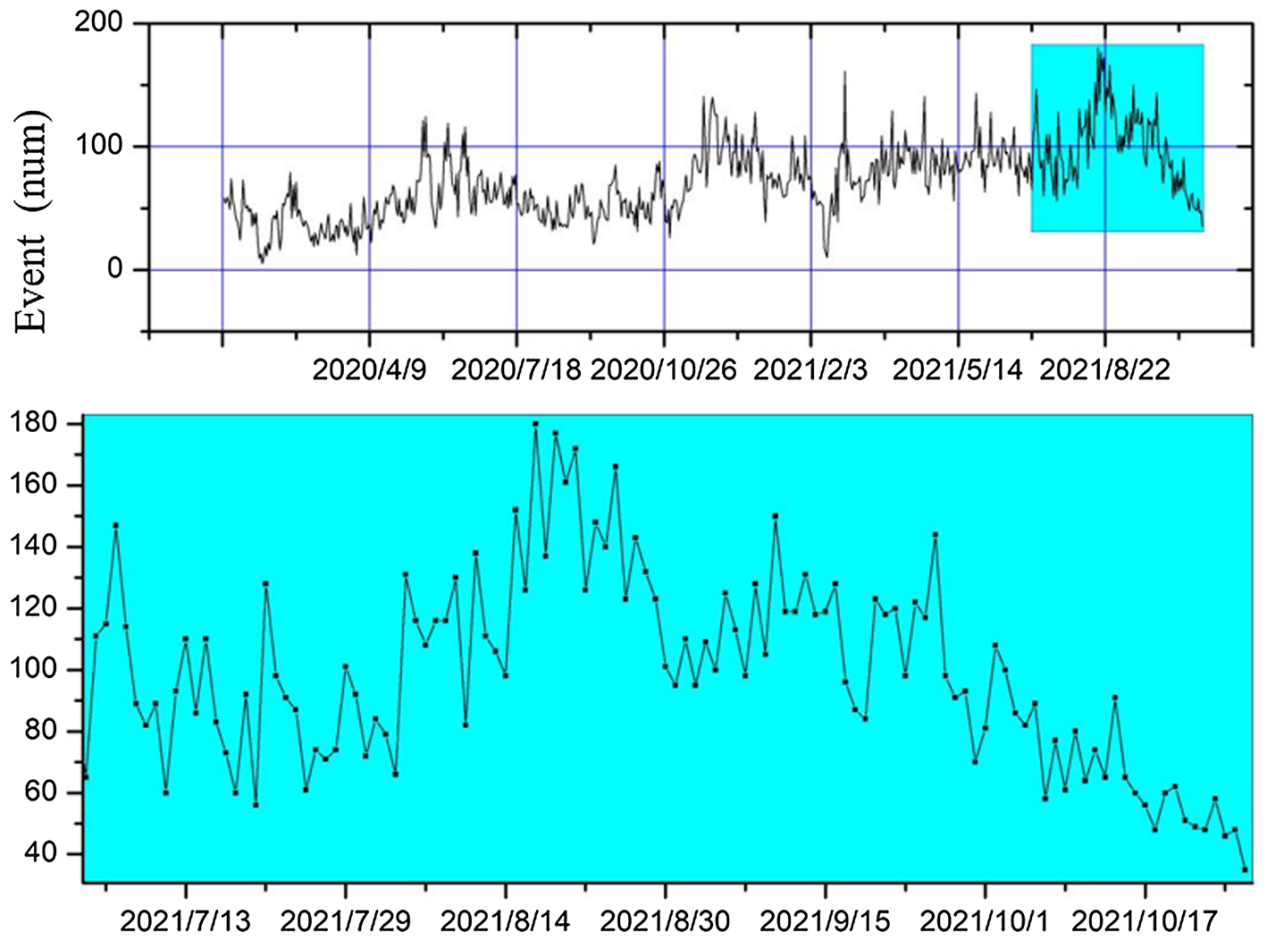
Time series of microseismic energy events in the Junde coal mine.

I combine the above analysis with the analysis from the temporal energy data of the working face in the Junde coal mine. The background value of energy events in the Junde coal mine is changing continuously since April 9, 2020. By observing the enlarged graph intercepted in Fig. 2, it can be found that the number of energy events shows a rapid increase from the highest daily occurrence of more than 100 energy events to more than 180 events starting from August 14, 2021. Meanwhile, the energy event sequence of the last 2 years shows 5 peaks, corresponding to February, June, November 2020, February, and August 2021, 4 of which are accompanied by the rock burst phenomenon.

At the same time, working face pressure monitoring was done. By keeping an eye on mine pressure monitoring data, it was found that mine pressure was revealed before each large energy event. Therefore, the author makes a time-series empirical analysis of long-term mine pressure data and finds that whenever a periodic large energy event occurs, the mine pressure will gradually increase before the cycle, and the phenomenon of mine pressure accumulation will occur before the next large energy event. A large amount of mine pressure data shows that the initial incoming pressure cycle at this working face is generally 2–3 days, and the periodic incoming pressure is generally around 5–10 days, with a general step of 10–25 m.

At the same time, the author also analyzed the long-term microseismic data of the Junde coal mine for the cycle evolution and combined it with the regularity of mine pressure manifestation, and concluded that the daily microseismic frequency increased 10 times continuously within 3–7 days, and the total microseismic energy increased continuously within 3–7 days with mine pressure manifestation. In this cycle, the average energy change deviates from the period of no impact pressure precursor, and the single-day microseismic frequency and total microseismic energy change abnormally, and the microseismic events gather in the local area within 3–7 days. The cycle evolution analysis reveals the changing pattern of rock burst microseismic event precursors in Junde Mine.

The frequency, maximum energy, and average energy before the occurrence of the rock burst have a significant deviation from the normal value of the changing pattern, and the most typical change lies in the energy change of the week before it. Given the above analysis and the processing of the expert system, the author constructed the "period-energy-frequency" model. The model will "noise reduce" the data. The model eliminates data that are not meaningful based on the pattern of microseismic events at the mine.

**Figure 3:**
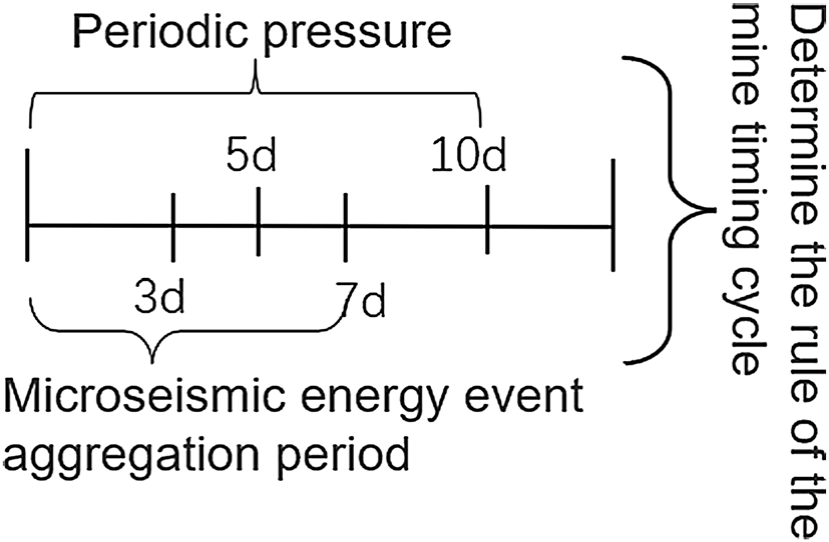
Mine time series cycle interval.

The construction of this model also provides a new way of thinking for the data processing of other mine safety testing equipment, as shown in Fig. 3. Figure 3 includes mine pressure cycles and microseismic energy event cycles, and different cycles were chosen based on the analysis of the corresponding data for this mine, with the microseismic labeled 3d and 7d, and the mine pressure labeled 5d and 10d, respectively.

The cumulative energy, average energy, and frequency of each seven days were used as input data for the neural network. This gives a total of 130 sets of data. The maximum energy event of the microearthquake will directly affect the mine safety, so the maximum energy can be taken as output data to form the prediction sample of the neural network.

This allows the data from the previous group to be used to predict the maximum energy of the next group of microseismic energy events. Due to the inconsistent units of the monitoring data, the variation of the magnitudes is large. If the data are fed into the model for training without any processing, there will be an impact on the fit of the model and thus on the accuracy of the model and also on the convergence rate of the model. Therefore, the original data are normalized^22^, thus avoiding the effects due to the different magnitudes of the data. As shown in Eq. 1.

1 $$ x^{\prime}_{i} = \frac{{x_{i} - x_{\min } }}{{x_{\max } - x_{\min } }} $$

where X_i_ and X_i_' are the original data and the normalized data, and X_max_ and X_min_ are the maximum and minimum values in the same component.

### MEA-BP neural network prediction steps

1. "Noise reduction" of the data using the "period-energy-frequency" model.
2. To avoid errors caused by different magnitudes, the data are normalized.
3. Determine the topology of the BP neural network, create the initial training set and test set, the corresponding initial population using the initial population generation function, and the winning sub-population and temporary sub-population using the sub-population generation function.
4. After the winning subpopulation and temporary subpopulation are generated, each subpopulation is subject to the convergence operation, and the population maturity discriminant function is used to determine whether the subpopulation "convergence" operation is completed. When the temporary subpopulation and the winning subpopulation are generated, each subpopulation must perform the "convergence" operation and use the population maturity discriminant function to determine whether the "convergence" operation of the subpopulation is completed.
5. The subpopulations are dissimilarities and new subpopulations are added according to the results of the dissimilarization operation^23^.
6. When the iteration is stopped, the MEA algorithm ends and the optimal individual is found and used as the weight and threshold of the BP neural network^24^.
7. In order to train and learn utilizing the training data that has already been processed, the resulting weights and thresholds are used as the BP neural network's initial weights and thresholds.
8. A neural network model that achieves the effect is created after the training is finished and tested.

**Figure 4:**
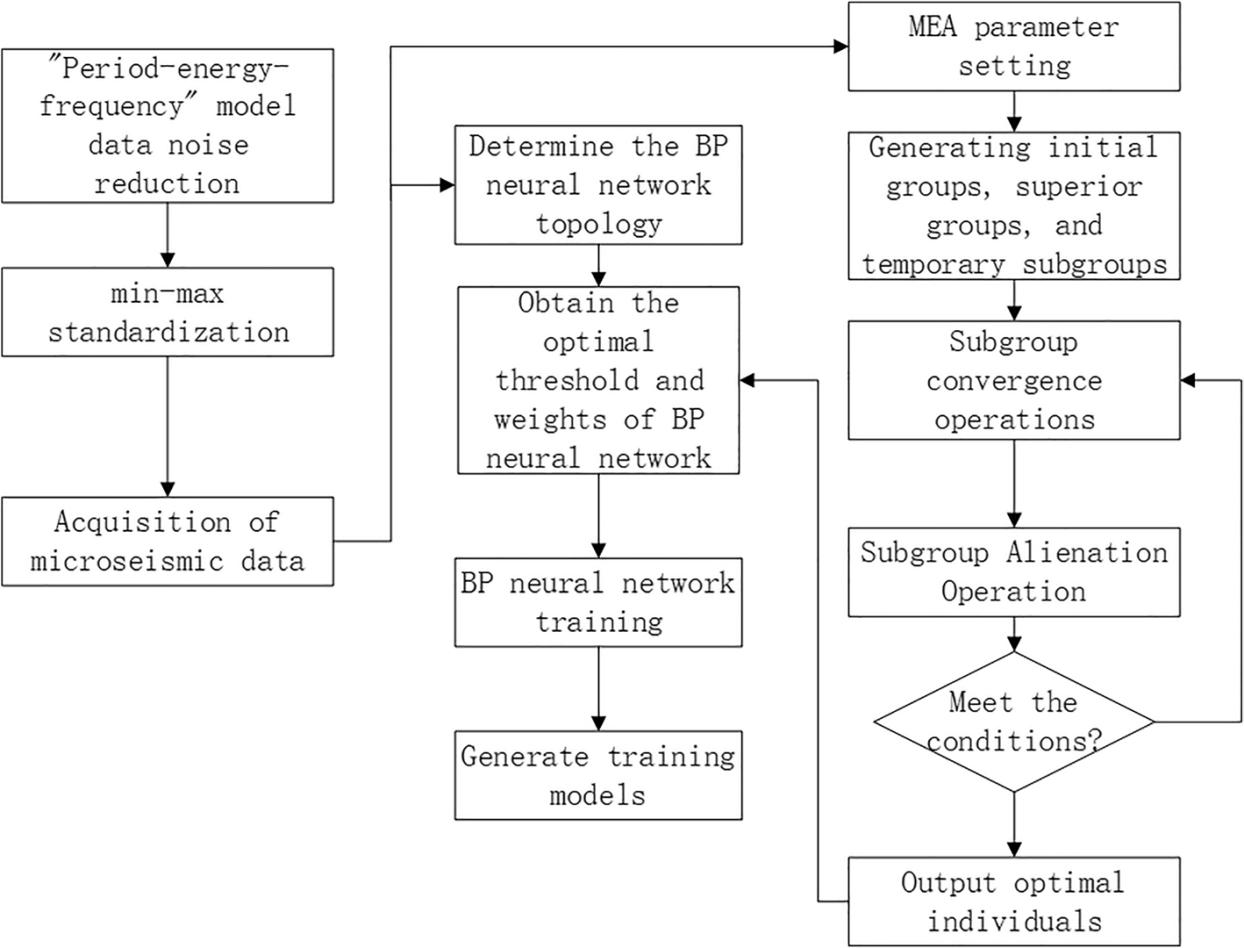
Prediction flow chart.

The prediction process is shown in Fig. 4.

### Data testing by applying the MEA-BP neural network model

The author initially sorted the gathered data in accordance with the algorithm prediction procedure and set the BP neural network's input layer nodes to three, output layer nodes to one, and hidden layer nodes to eight, seven, and six. Finally, the number of hidden layer nodes is fixed at 7 based on the various experimental findings^25^.

The parameters of the MEA algorithm were set according to the structural characteristics of the system, the population size was set to 300 according to the continuous debugging effect, the number of superior and temporary subpopulations was set to 5^26^, and the parameters S1, S3, and S2 of the input layer, output layer^27^, and hidden layer were set to 3, 7, and 1, respectively^28,29^. After continuous operations, the optimal weights of the BP neural network and the threshold values were obtained. Then, the data are assigned to the corresponding parameters and trained using the training data that have been initialized. After the training is completed, the test data is used for testing. All the above operations are done by MatlabR2020a.

## Analysis of MEA-BP microseismic prediction results

According to the above study, 125 sets of data were selected as training data and actual monitoring of the mine for 35 days as validation data, to test the actual prediction effect. Two neural networks, MEA-BP neural network, and BP neural network were used for the experiments respectively, and run by MatlabR2020a, by comparing the performance of the MEA-BP neural network and BP neural network methods and the prediction effect. From these two neural networks, the prediction outcomes, convergence speed, and degree of data fit were examined; the mean square error curve analysis revealed the model's best validation performance and convergence speed. The degree of fitting of the two neural networks to the data may be determined by examining the correlation coefficient R of the neural network regression curve, and the relative error and absolute error of the two neural networks can be compared to evaluate the prediction effect.

**Figure 5:**
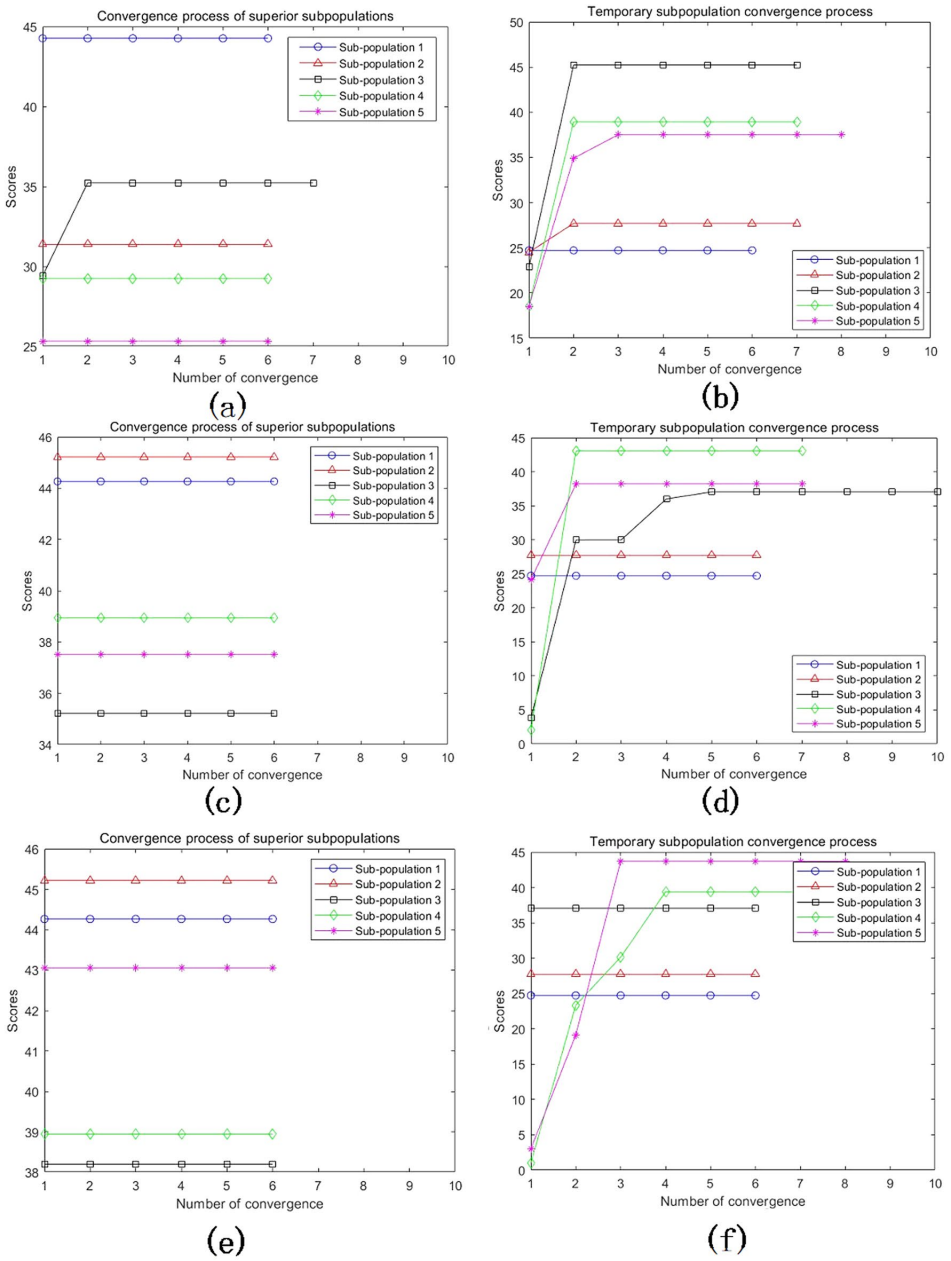

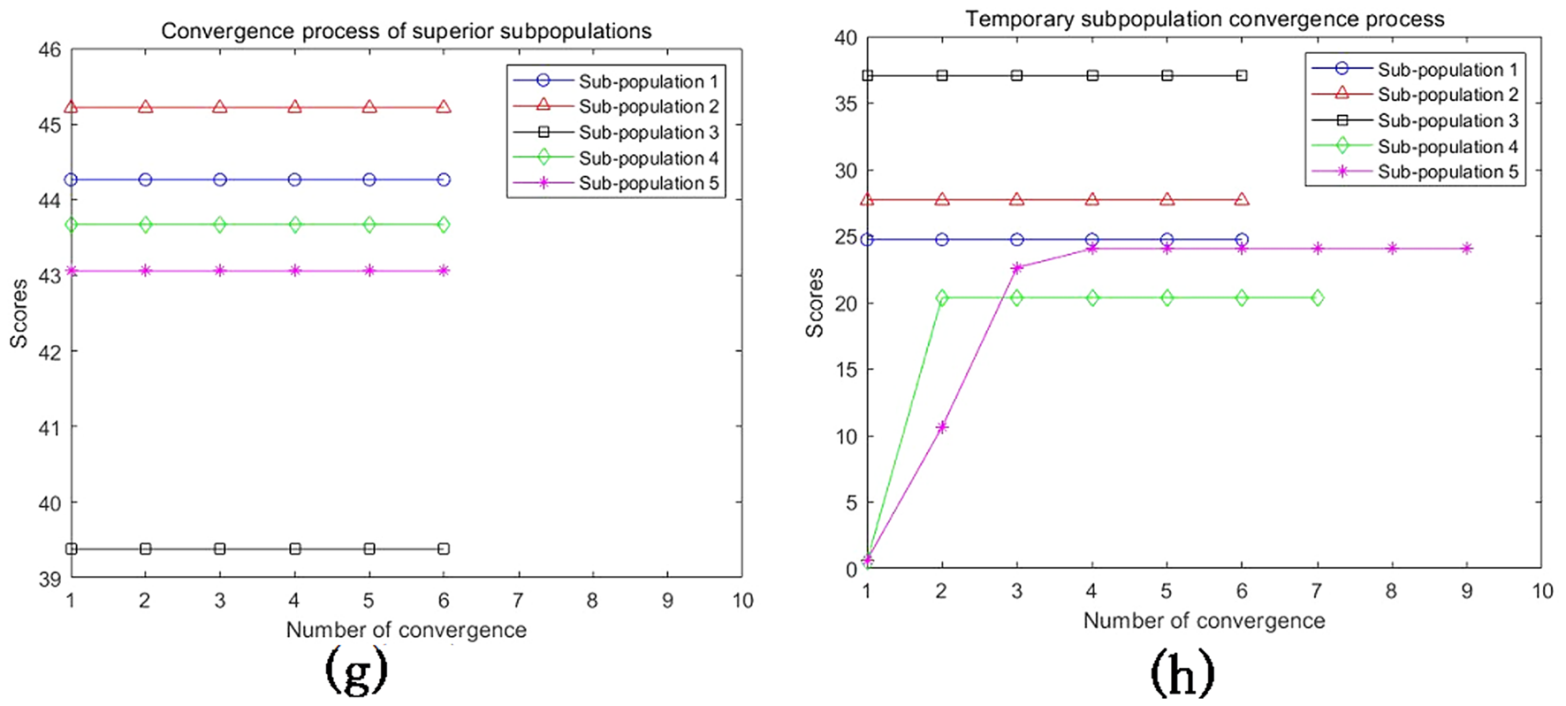
Convergence diagram of the subpopulation.

**Figure 6:**
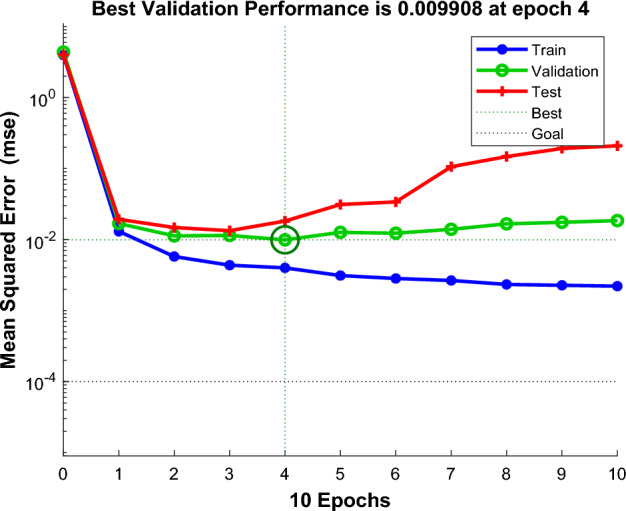
BP neural network regression state curve.

**Figure 7:**
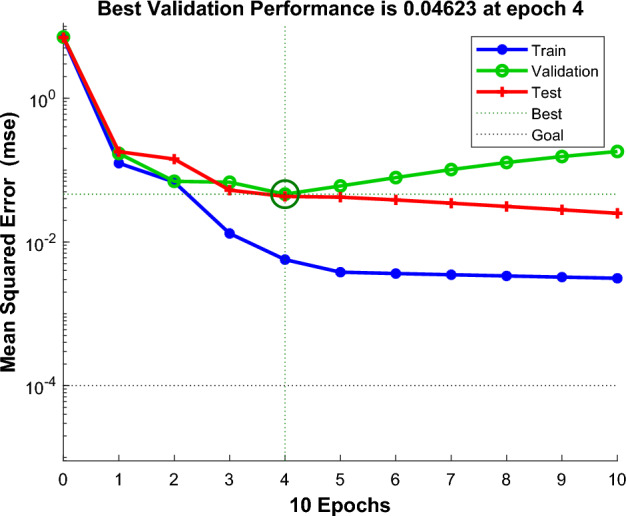
Regression state curve of MEA-BP neural network.

**Figure 8:**
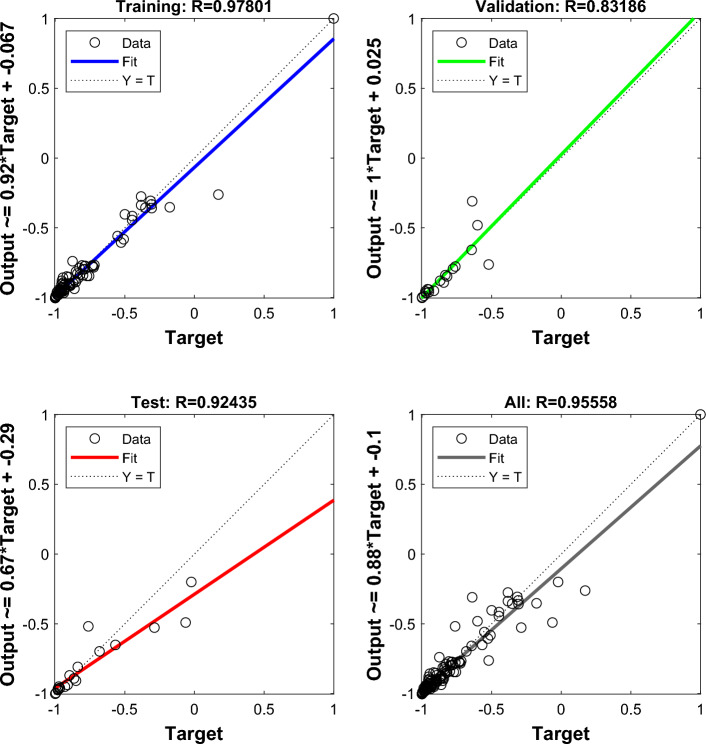
MEA-BP neural network regression state.

**Figure 9:**
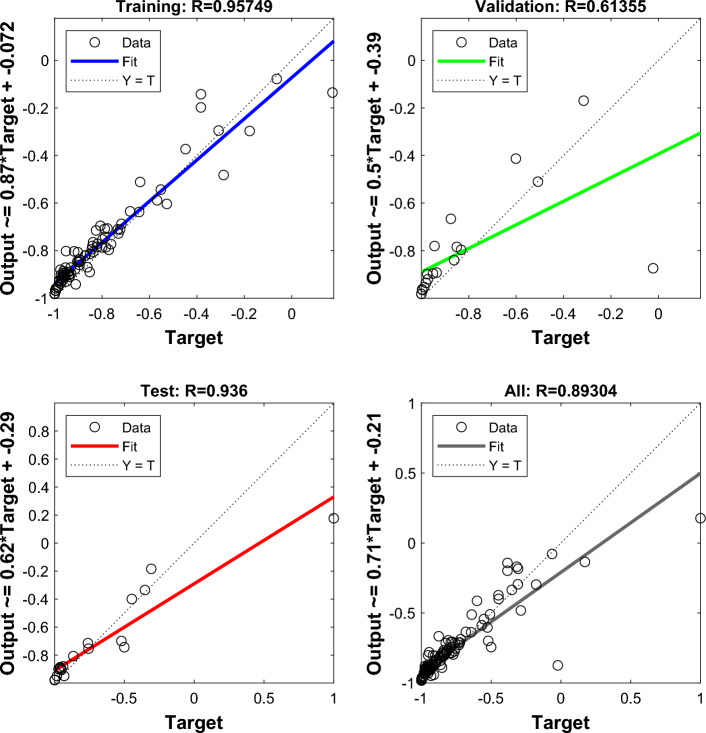
Regression state of BP neural network.

**Figure 10:**
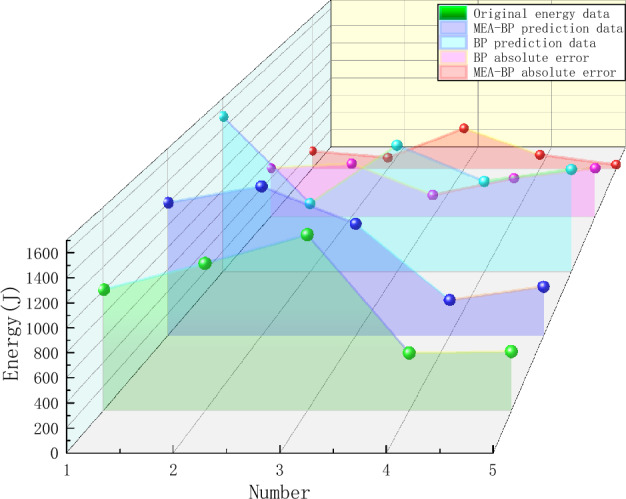
Absolute error contrast.

**Figure 11:**
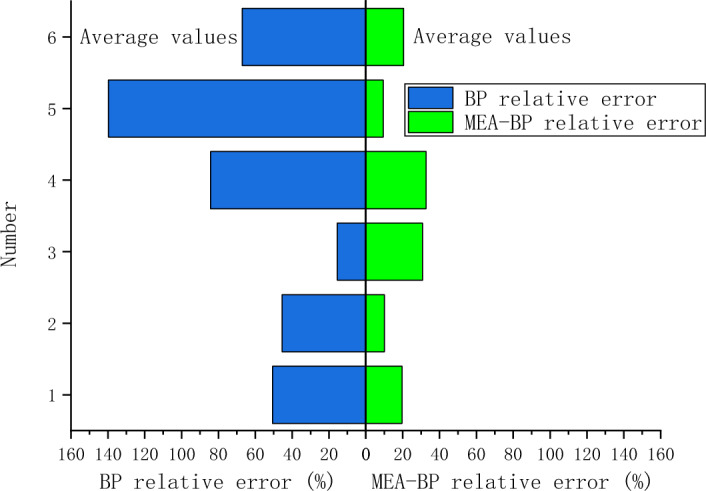
Relative error contrast.

1. The "convergence" process of MEA algorithm optimization can be obtained by observing the subpopulation convergence process in Fig. 5. It can be found that after multiple "convergence" operations, subpopulation 3 in the temporary subpopulation in Fig. 5b still has a higher score than subpopulation 3 in the winning subpopulation in Fig. 5a, and the MEA algorithm still needs to perform one more "dissimilation" operation, and at the same time, a population needs to be added to the temporary population to perform the "convergence" operation again, and after the operation is completed, it is found that the temporary sub-population still has a higher score than the winning sub-population. Therefore, the above operation will be continued, and the subpopulation will be matured by repeating the above process.
2. The mean square error (MSE) curve is an indicator of the neural network's ability to anticipate outcomes and rate of convergence. The difference between the projected value and the actual value, squared, is what is referred to as the mean square error. When the MSE number is lower, it can denote a more accurate forecast in light of the pertinent circumstances. The MSE can reflect the fluctuation in the data. The MSE error curve in Fig. 6 shows that the best validation performance of the BP neural network is 0.009908, and it has started to converge after 4 training sessions; the mean square error curve in Fig. 7 shows that the best validation performance of the MEA-BP neural network is 0.04623, and its data training set also starts to converge after 4 training sessions, which fully indicates that the MEA-optimized BP neural network has better accuracy than BP neural network has better accuracy.
3. The correlation coefficient R is mainly used to judge the good or bad situation of the fitted data of the neural network. When the neural network fits better, its R-value is closer to 1, otherwise, it means that the neural network fits poorly. The regression state curve of the BP neural network and the regression state curve of the MEA-BP neural network are shown in Figs. 8 and 9. The training set, validation set, test set, and all the correlation coefficients of the MEA-BP neural network are 0.97801, 0.83186, 0.92435, 0.95558, and the experimental parameters of the BP neural network are 0.95749, 0.61335, 0.93600, 0.89304. It can be seen that the correlation coefficient of the MEA-BP neural network is closer to 1 than that of the BP neural network, and its fitting degree of data is better. According to the analysis of the experimental results, the MEA-BP neural network did not show any overfitting.
4. As seen in Figs. 10 and 11, the MEA-BP neural network performs significantly better than the BP neural network, with the maximum absolute error and maximum relative error of the BP neural network being 559.3 J and 139.7%, respectively, and 453.8 J and 32.7%, respectively, for the MEA-BP neural network.
5. The comparison of the MEA-BP neural network and the BP neural network is presented in Figs. 10 and 11. It is clear that the MEA-BP neural network's prediction error has a lesser overall trend than the BP neural network. Additionally, it is known that the average absolute error and relative error of the MEA-BP neural network are 196.34 J and 20.5%, respectively, and that the average absolute difference and average absolute error of the BP neural network are 443.58 J and 67.1%, respectively. The accuracy of the MEA-BP neural network prediction is higher when compared to the findings of the BP neural network prediction. The MEA algorithm helps the BP neural network perform better.

## Validation of results

**Figure 12:**
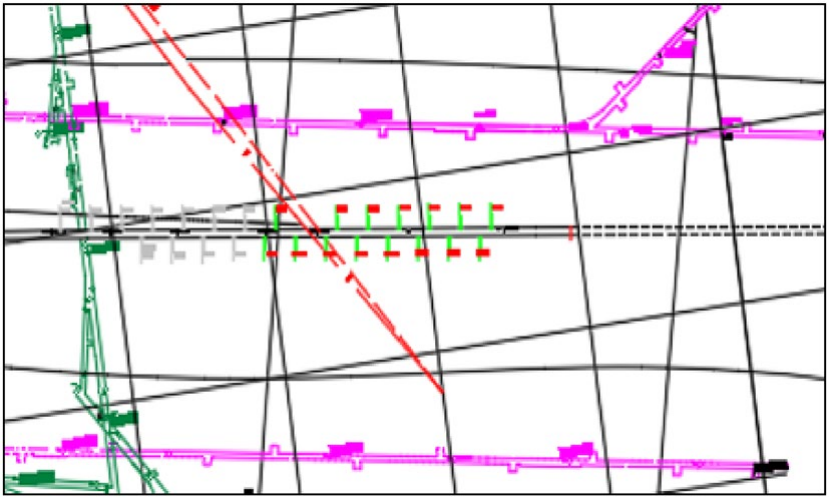
KJ550 rock explosion online monitoring system layout.

To further verify the accuracy of the MEA-BP neural network prediction, the KJ550 rock burst online monitoring system is used as shown in Fig. 12.

Stress sensors are installed in the upper and lower gang of the duct and in the coal body of the upper and lower gang of the machine. The location of the hole opening in the upper gang of the air duct is 1 ~ 1.5 m from the bottom plate, the height of the hole opening in the lower gang of the air duct is 0.5 ~ 1 m from the bottom plate, the height of the hole opening in the upper gang of the machine duct is 1.5 ~ 2 m from the bottom plate, and the height of the hole opening in the lower gang of the machine duct is 0.5 ~ 1 m from the bottom plate. The installation depth of the stress gauge is 8 m for shallow holes and 14 m for deep holes, with a spacing of 1 m. The stress gauge pressure should be filled with oil to make up the pressure when it is lower than 3 MPa. The yellow warning value for shallow holes is 10 MPa; the red warning value is 12 MPa; the yellow warning value for deep holes is 13 MPa; the red warning value is 15 MPa.

The KJ550 monitoring system will monitor the changing pattern of the mining stress field at the working face, and the two indicators of stress and stress change rate will be used to determine the impact hazard at the monitoring point. According to the model of the "period-energy-frequency" prediction method, the prediction period of the MEA-BP neural network is 7 days. Therefore, the author uses the stress and maximum stress rate of change every seven days as the corresponding verification of the prediction results.

The maximum stress and maximum stress increment of the corresponding five groups did not exceed the alert value over the course of 35 days of continuous online stress monitoring. Additionally, the maximum stress of each group did not exceed 10 MPa, and the maximum 24-h stress change rate of each group did not exceed 1.5 MPa. The stress monitoring findings and the anticipated energy events lined up, further confirming the accuracy of the forecast results.

## Conclusion

Through the analysis of microseismic data and mine pressure data evolution of the working face of the Junde coal mine, it is concluded that the frequency, maximum energy, and average energy before the occurrence of rock burst tend to deviate significantly from the normal value, and there is a mine pressure manifestation, and the most typical change is the energy change in the previous week. The "cycle–energy–frequency" data processing model is constructed. The MEA-BP neural network conducts validation better than the BP neural network, and its correlation coefficient R is nearer 1. The MEA-BP neural network prediction result's absolute error is reduced by 247.24 J, and the relative error is decreased by 46.6%. The better fitting effect, smaller prediction error, and improved prediction accuracy are all characteristics of the MEA-BP neural network. Meanwhile, according to the analysis of the prediction results, no overfitting of the model occurred. It shows how the MEA-BP neural network can be used to forecast microseismic activity in the Junde coal mine. The MEA-BP neural network prediction results have an absolute average error of 196.34 J and an average relative error of 20.5%. The working face was continually observed by the stress online monitoring system, and the results of the stress monitoring matched those of the microseismic energy event forecast. MEA-BP neural network can better predict the magnitude of microseismic energy events in Junde Coal Mine, which has reference value for predicting microseismic events in rock burst mines in Junde Coal Mine. It can provide strong support for the prevention and control of rock burst.

## Data Availability

The datasets used and analyzed during the current study are available from the corresponding author upon reasonable request.
